# 1600 Parallel Microchamber Microfluidic Device for Fast Sample Array Preparation Using the Immiscibility of Two Liquids

**DOI:** 10.3390/mi8030063

**Published:** 2017-02-23

**Authors:** Chul Min Kim, Gyu Man Kim

**Affiliations:** School of Mechanical Engineering, Kyungpook National University, 80 Daehakro, Bukgu, Daegu 41566, Korea; faithfulsaint@daum.net

**Keywords:** sample array, microfluidics, parallel microfluidic device, high throughput

## Abstract

We present a 1600 parallel microchamber microfluidic device for fast sample array preparation using the immiscibility of two liquids. The trapping efficiency and size of the arrayed sample in the microchambers of a parallel microfluidic device were analyzed at various flow rates. The trapping efficiency of the sample was also inspected according to the position of the microchamber. Samples were successfully arrayed using the device. The trapping efficiency of the sample was 98.69% at 1 mL/h. The trapping efficiency and diameter of the sample decreased as the flow rate increased. Trapping efficiencies also changed according to the position of the microchambers. As the distance of the microchambers from the inlet increased, the sample trapping efficiency decreased. This tendency occurred more clearly at higher flow rates.

## 1. Introduction

Over the past decade, high-throughput screening (HTS) has been developed for various applications related to biology, pharmacy, chemistry, and medicine [1,2,3]. Higher performance is being demanded by a growing number of applications because of advancements in biology, genetics, and genomics [4]. Because conventional screening is labor-intensive and time consuming, most HTS applications employ robotic systems to avoid human error [5]. However, improvement in current HTS methods is required because of scientific developments and financial pressure [6]. There are several reasons for the decline in the use and development of HTS applications. First, robotic systems for HTS are expensive, especially for small laboratories in universities [7]. Second, the cost of reagent target samples is high [8]. Furthermore, it is difficult for robotic systems to reduce the volume of a sample to less than 1 µL because of the uncontrollable evaporation of liquid solutions [9].

Sample arrays based on droplet microfluidic systems can supply physical isolation of a sample from contamination by surrounding the sample with an immiscible phase [10,11]. Additionally, fast and efficient reaction of reagents can be implemented because of reduced volume (1 pL−10 nL). Because arrayed droplets can be stably stored after trapping, liquid solutions of a reagent and a target can be re-introduced into a microfluidic system for post-processing [12]. Related research has been conducted by many groups: Sun et al., demonstrated that arrayed droplets in a static droplet array have gradients of concentration [13]; Cohen et al., showed that droplets can be arrayed by a static droplet array using the immiscibility of two liquid phases [14]; Gansen et al., proposed a digital lamp that uses two immiscible fluids with fluidic force and interfacial tension [15]; and Xu et al., studied a droplet-clustering array device with a guide track to guide droplets into the physical compartment [16]. However, most of the microfluidic devices of these studies were designed with a single channel, which limits the throughput to establish a sample array. Throughput can be increased by adopting multi-channels in the microfluidic device, where sample droplets are stored simultaneously in each channel [17]. The parallel process of a multi-channel microfluidic device improves the throughput with a simple process at a low analysis cost. Our group has proposed a parallel microfluidic device of 108 microchambers [18]. However, in order to satisfy requirement from various field, it is necessary to fabricate a microfluidic device with increased number of microchabmers that can accommodate more than 108 samples. Furthermore, with an increasing number of microchamber, trapping efficiency should be analyzed according to various conditions such as location and flow rate.

In this study, we propose an extended parallel microfluidic device from 108 to 1600 microchambers for fast sample array preparation using the immiscibility of two liquid phases. Higher parallelization, with more individual chambers than previous work, can be conducted than previous work. The proposed device estimates the sample trapping efficiency and the diameter of the trapped sample according to various flow rates. Behaviors of the sample array at various locations are inspected.

## 2. Materials and Methods

### 2.1. Sample Array Process in Parallel Microfluidic Device

**Figure 1:**
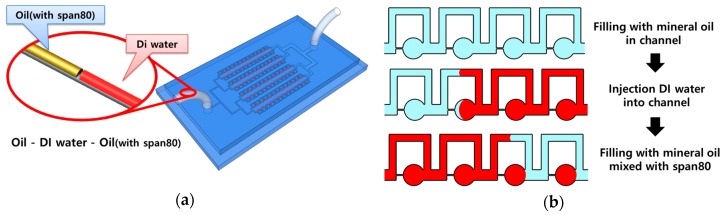
Schematic (**a**) and sample array process (**b**) of parallel microfluidic device.

Figure 1 shows the schematic and sample array process of the parallel microfluidic device. Three liquids (mineral oil, DI water, and mineral oil mixed with Span 80 (0.1% *w*/*v*)) were sequentially injected into the parallel microfluidic device at a constant flow rate using a syringe pump. To array the sample using the parallel microfluidic device, the channel was filled with mineral oil and then with DI water. Finally, mineral oil mixed with Span 80 was injected into the channel. DI water was used as the sample to be arrayed in the microchambers of the device. A rhodamine dye was mixed with the DI water to enhance observation. Mineral oil was used to isolate the DI water in the microchamber. Span 80 was added to the mineral oil to create a stable sample array by reducing the interfacial tension of the water droplets. The arrayed samples of 1600 microchambers were captured and measured by an inverted microscope and image capture software. Experiments were conducted in triplicate.

### 2.2. Design of Parallel Microfluidic Device

The parallel microfluidic device consisted of a microchamber, an orifice channel, and a bypass channel. The device had 32 micro channels placed in parallel with 50 microchambers in each channel. A total of 1600 isolated sample droplets could be trapped in the microchambers. The width of the main channel was 150 µm, and the diameter of the microchamber was 300 µm. The length and width of the orifice channel were 100 and 15 µm, respectively. The width and length of the bypass channel (BW, BL) were 100 and 1150 µm, respectively. In prior research, the geometry of the parallel microfluidic device was determined by computer simulation (COMSOL) [18]. Six simulation models were selected according to various sizes of BW (750, 950 and 1150 µm) and BL (100 and 150 µm). The simulation was conducted in two dimensional, non-slip state and time-dependent. Used flow rate of simulation at inlet was 0.4 mL/s. Flow rate in orifice changed with BL and BW. Flow rate in orifice decreases with increasing of BW because flow resistance in bypass channel decrease. When BW was 100 µm, the flow rate in orifice increase as the BL increased due to increase of flow resistance in bypass channel. After COMSOL simulation, six types of microfluidic devices were tested for water sample array. When BW was 150 µm, Water did not trap in microchamber and pass through bypass channel because of high flow resistance at orifice. By contrast, When BW was 100 µm and BLs were 750 and 950 µm, Water was unstably trapped in microchamber or squeezed out from orifice due to higher flow rate with low flow resistance in orifice. Therefore, samples can be isolated most stably in microchambers when BW and BL were 100 and 1150 µm, respectively.

### 2.3. Fabrication of Parallel Microfluidic Device

**Figure 2:**
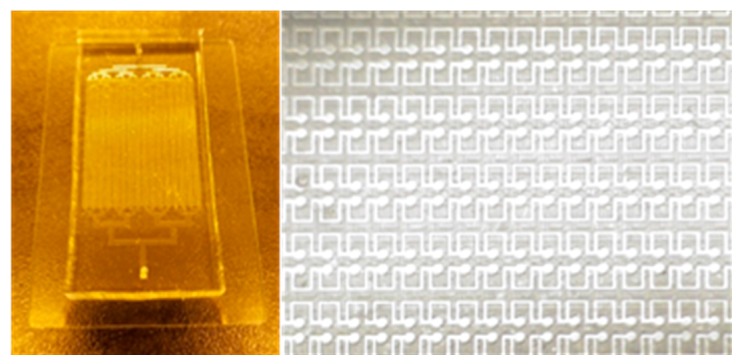
Optical images of fabricated parallel microfluidic device.

The master mold of the parallel microfluidic device was fabricated by conventional photolithography using SU-8 2100 negative photoresist. The thickness of the master mold was 100 µm, and the parallel microfluidic device was replicated from the master mold by using conventional polydimethylsiloxane (PDMS) casting. The PDMS prepolymer solution was mixed with curing solution at a 10:1 ratio. The resulting solution was poured into the master mold and placed in a vacuum chamber for 30 min to remove any trapped bubbles and cured in an oven at 90 °C for at least 2 h. The cured PDMS replica was separated from the master mold and punched with a 1.5-mm-diameter puncher to make inlets and outlets. The parallel microfluidic device was permanently bonded with slide glass by an oxygen plasma treatment. The fabricated device was placed in an oven overnight at 90 °C to recover the hydrophobicity of the microchannels. Figure 2 shows the optical images of the fabricated 1600 parallel microchamber microfluidic device.

## 3. Results and Discussion

### 3.1. Behavior of Sample Array According to Flow Rate

**Figure 3:**
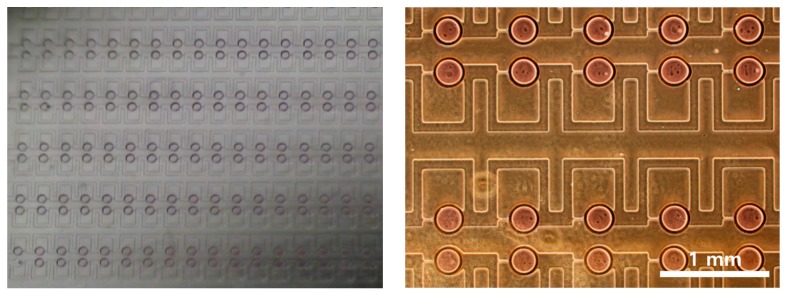
Optical images of arrayed water sample using parallel microfluidic device.

**Figure 4:**
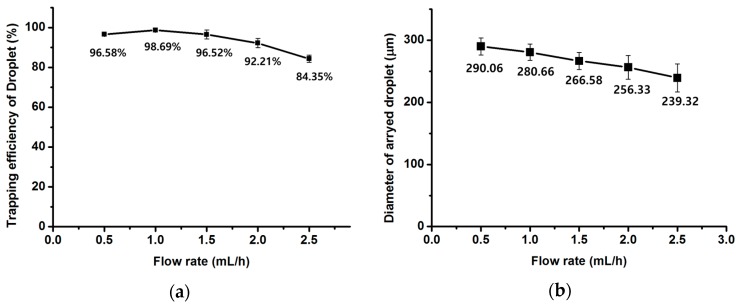
Trapping efficiency (**a**) and diameter (**b**) of water sample array according to flow rates.

Figure 3 shows optical images of the arrayed sample in the microchambers of the microfluidic device. The sample solution could be stably trapped in the microchambers. Figure 4 shows the trapping efficiency and the diameter of the trapped water sample according to flow rates. The trapping efficiency was approximately 98.69% at 1 mL/h. Trapping efficiencies exceeded 90% at flow rates from 0.5 to 2.0 mL/h. As flow rates increased, trapping efficiencies gradually decreased. When the flow rate reached 2.5 mL/h, the trapping efficiency decreased to 84.35%.

**Figure 5:**
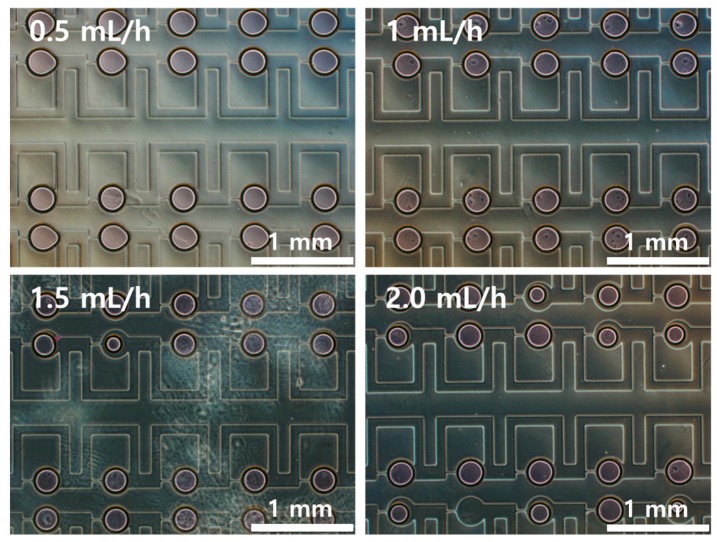
Optical images of arrayed sample in parallel microfluidic device at various flow rates.

Because the time for the full trapping of the sample in the microchambers was insufficient at higher flow rates, partially trapped samples could not be spilt from the sample flow in the bypass channel and were withdrawn from the microchambers to the bypass channel again. As flow rate increased, the diameter of the arrayed sample in the microchambers gradually decreased because more sample solution was separated from the trapped sample by the increased shear force at higher flow rates as shown in Figure 5. When flow rates increased from 0.5 mL/h to 2.5 mL/h, the diameter of the sample in the microchambers decreased by approximately 18%.

### 3.2. Behavior of Sample Array According to Microchamber Position

**Figure 6:**
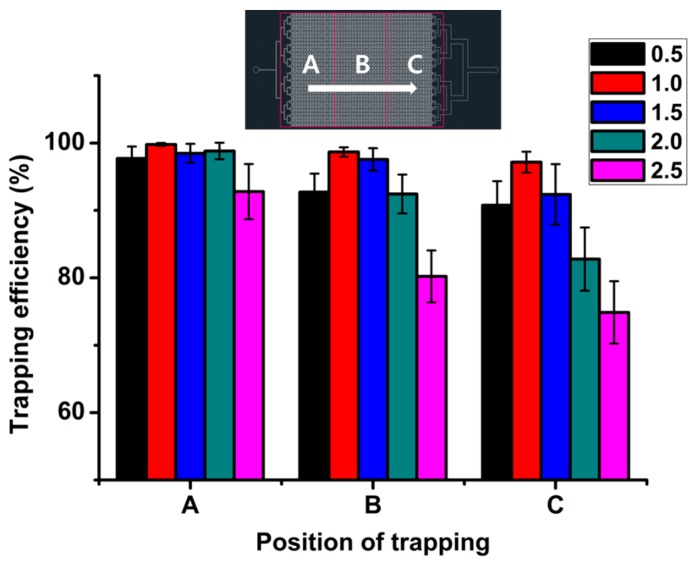
Trapping efficiency of sample in parallel microfluidic device according to various positions.

Trapping efficiencies were analyzed according to the position of the microchambers in the microfluidic device. Figure 6 shows the trapping efficiency according to the position of the microchamber. The microchannel was divided into three observation areas for analysis: A, front; B, middle; and C, back. The sample arrays in each area of the 160 microchambers were observed. There was no significant difference in trapping efficiency at a flow rate of 1 mL/h. However, the trapping efficiency decreased in the C area as flow rates increased to 2.5 mL/h. Since the fluid in the orifice channel could not flow because of the trapped sample in the microchamber, the flow rate in the bypass channel increased and the trapping efficiency in the C area decreased. There were no significant differences in the diameter of the trapped sample according to the position of the microchamber. The effect of microchamber position was also inspected at the right, middle, and left areas. The trapping efficiency of the sample in the middle area was slightly higher than that in the side areas, but was not sufficiently significant to explain the effect of the symmetry of the positions.

## 4. Conclusions

We presented a 1600 parallel microchamber microfluidic device for fast sample array preparation. The sample array in the microchambers of the fabricated device was analyzed at various flow rates. Stability and repeatability of the arrayed sample in the microchambers were demonstrated by a water/oil test. As a result, the trapping efficiency and diameter of the trapped sample were 98.69% and 280.66 µm at 1 mL/h, respectively. The trapping efficiency and diameter of the sample in the microchambers decreased as the flow rate increased because of the shear force of the flow around the trapped sample. The trapping efficiency of the sample in the front area of a microchannel was higher than that in the rear area. This phenomenon occurred more clearly at higher flow rates. The trapping efficiency of the sample in the middle area was slightly higher than that in side areas, but there was no significant difference. This device can be used as an inexpensive effective tool for fast sample array preparation for drug screening and various applications in the fields of cytotoxicity and biology.
